# Reversible Acute Kidney Injury Associated with Chlorothalonil Ingestion

**DOI:** 10.5811/cpcem.2017.6.34722

**Published:** 2017-10-03

**Authors:** Jeffrey R. Suchard

**Affiliations:** University of California Irvine, School of Medicine, UC Irvine Health, UCI Medical Center, Department of Emergency Medicine and Pharmacology, Orange, California

## Abstract

A 43-year-old man ingested a chlorothalonil-containing fungicide in a suicide attempt. The patient was found to have acute kidney injury from acute tubular necrosis on hospital admission (serum creatinine 2.9 mg/dL), although his renal function recovered with hydration and supportive care. Acute toxicity from chlorothalonil ingestion has been described very rarely, and no previous cases have described clinically-significant renal effects.

## INTRODUCTION

Chlorothalonil is a fungicide commonly used in agriculture and horticulture.^1^,^2^ Chronic workplace exposure to chlorothalonil is known to potentially cause pulmonary and dermatologic sensitization.^3^–^8^ Reports of acute human toxicity are very rare and have not included clinically-significant renal effects.^9^–^10^ In the case reported here, intentional ingestion of a commercially-available, chlorothalonil-containing fungicide resulted in reversible acute kidney injury.

## CASE REPORT

A 43-year-old man presented to the emergency department (ED) by ambulance 12 hours after intentionally ingesting an estimated eight fluid ounces of Daconil® fungicide concentrate (29.6% chlorothalonil) and 16 ounces of a domestic multi-surface cleaner (Mr. Clean® with Febreze Freshness Antibacterial Spray; primary active ingredient ≤5% 3-butoxypropan-2-ol). The patient reported 5–6 episodes of vomiting and diarrhea since the ingestion, which was a suicide attempt in response to persecutory delusions. He had a history of alcohol abuse and seizures, and had been diagnosed with an unspecified psychotic disorder during previous hospitalizations. The patient had undergone a craniotomy to decompress a subdural hematoma one month prior to the current visit and was readmitted after another seizure two weeks later, but had not required further surgical intervention.

On initial evaluation in the ED, the patient was moderately tachycardic (118 beats/min) and hypertensive (147/92 mmHg) although the physical examination, including the oral cavity and abdomen, was unremarkable. Laboratory evaluation revealed serum blood urea nitrogen (BUN) of 22 mg/dL and a serum creatinine of 2.9 mg/dL. Lab results from two weeks previously had shown a BUN of 11 mg/dL and a creatinine of 0.9 mg/dL. The serum anion gap was normal at 11 mEq/L, lactate was 1.6 mEq/L, calcium was 11.1 mg/dL, and creatine phosphokinase was mildly elevated at 394 IU/mL. Serum osmolality was 295 mOsm/kg, representing an osmolal gap of approximately 11 mOsm/kg. The patient had serum levels of ethanol, acetaminophen, and salicylate below the lab’s detection limits. A serum volatiles test detected no methanol or isopropanol to account for the patient’s osmolal gap. A urine drugs-of-abuse screen was positive only for benzodiazepines (negative for stimulants: amphetamines as a class, methylenedioxymethamphetamine, and cocaine). Urinalysis with microscopy showed 47 muddy-brown granular casts per low-power field, consistent with acute tubular necrosis; there was no crystalluria. The patient was diagnosed with acute kidney injury and consultations were made with nephrology, psychiatry, and toxicology.

The patient was treated initially with ondansetron and three liters intravenous (IV) 0.9% saline, and admitted to the internal medicine service. Neither nephrology nor toxicology recommended renal replacement therapy (dialysis) at the time of admission, recommending instead hydration, supportive care, and monitoring renal function through serial labs and urine output. Orders were submitted to the laboratory to obtain quantitative chlorothalonil levels in the patient’s serum and urine. The clinical pathologist on duty attempted to determine where such tests might be performed; however, no reference clinical laboratory could be identified to provide these services.

Repeat labs six hours after ED arrival showed BUN 23 mg/dL and creatinine 1.7 mg/dL, and these continued to decrease toward normal. On the morning of hospital day four, the serum BUN and creatinine were 7 mg/dL and 0.6 mg/dL respectively, and the patient was deemed medically stable for transfer to inpatient psychiatric care. The patient was discharged home on hospital day eight, having been restarted on phenytoin for seizures and risperidone for his psychotic symptoms. The patient was re-admitted five weeks later after ingesting a different household cleaning product, and was stable for further inpatient psychiatric care five days later.

## DISCUSSION

Chlorothalonil [see Figure] is a broad-spectrum organochlorine fungicide widely used in agriculture and horticulture.^1^,^2^ The mechanism of action appears to be inactivation of fungal enzymes containing sulfhydryl groups and depletion of glutathione. Chlorothalonil is, however, considered practically non-toxic in mammals, with a rat LD_50_ (lethal dose 50%) greater than10,000 mg/kg.^1^ Most of the human medical literature regarding chlorothalonil relates to low-grade, chronic exposures as would be encountered in workplaces where the fungicide is used. In these settings, chlorothalonil may cause dermatologic and respiratory sensitization effects, including occupational asthma, allergic contact dermatitis, contact urticaria, erythema dyschromicum perstans (ashy dermatitis), and anaphylaxis.^3^–^8^

Renal effects of chlorothalonil, but not renal failure, have been demonstrated in animal studies to determine consequences of long-term exposure, including carcinogenicity. Chronic exposure in rats results in proximal convoluted tubule hyperplasia and increased kidney weight; neither acute tubular necrosis nor kidney failure were reported.^11^ Chlorothalonil is considered possibly carcinogenic to humans (International Agency for Research on Cancer category 2B) based on evidence of carcinogenicity in animals, although human carcinogenicity has not been demonstrated.^2^

Previous reports of acute human chlorothalonil toxicity are very limited, and are not associated with kidney injury, as occurred in this case. Eight patients with acute chlorothalonil poisoning have been reported, only six of whom ingested the fungicide.^9^,^10^ A case series from Sri Lanka reported six patients with intentional chlorothalonil ingestions (and one with a mild inhalational injury).^9^ The six patients ingesting chlorothalonil developed a burning sensation of the mouth, throat, and epigastrium, and had dysphagia and vomiting. One patient had mild oral ulceration, and another had a single self-limited seizure. All six patients received supportive care only, and the median hospital stay was two days; no renal toxicity was mentioned. The authors concluded that human toxicity appears to be mild.^9^

**CPC-EM Capsule**

What do we already know about this clinical entity?

Chlorothalonil is a fungicide associated with dermatologic and pulmonary sensitization from chronic workplace exposure. Reports of acute human toxicity are rare.

What makes this presentation of disease reportable?

Ingestion of chlorothalonil was associated with reversible acute kidney injury in this patient, which has not previously been reported.

What is the major learning point?

In addition to seizure, gastrointestinal distress, and oral ulceration, acute chlorothalonil toxicity in humans may be associated with acute kidney injury.

How might this improve emergency medicine practice?

Intravenous hydration therapy and adequate supportive care can reverse acute kidney injury from several causes, including chlorothalonil ingestion.

One other case report suggested a link between inhalational exposure to chlorothalonil and development of diabetic ketoacidosis (DKA) two months later. Elevated chlorothalonil levels were confirmed in this patient’s serum and urine. The authors suggested that since chlorothalonil has been reported with “endocrine-disrupting” effects, this was a potential mechanism for inducing pancreatic beta-islet cell dysfunction. The only renal-related effect mentioned in this case report was the presence of intense ketonuria, as expected with DKA; serum markers of kidney function (BUN, creatinine) were not reported.^10^

The other household cleaning product the patient ingested is virtually non-toxic, and would not be expected to have caused any kidney injury; 3-Butoxypropan-2-ol was the primary active ingredient in the other product ingested. This compound (Chemical Abstracts Service 5131-66-8) is also known as butoxypropanol or propylene glycol n-butyl ether, and is a common ingredient in household and industrial cleaners where it helps solubilize hydrophobic greases and oils. The product’s Safety Data Sheet indicates that it may cause skin and eye irritation, but that it has no known toxicological effect on ingestion, and the only potential medical treatment indicated would be symptomatic care.^12^

As a relatively small, uncharged, water-soluble molecule, butoxypropanol could have affected the patient’s serum osmolal gap, which was mildly elevated (~11 mOsm/kg) on initial lab testing. The concern here is whether the butoxypropanol could account for the entire osmolal gap, or whether the osmolal gap and renal injury may have occurred due to unreported ethylene glycol ingestion, which would warrant specific treatment. If the estimated amount of the cleaning product ingested were correct, and that product contained 5% butoxypropanol (upper limit listed on the SDS), then the total amount consumed would be around 24 mL, which corresponds to ~21 g. If this entire amount of butoxypropanol were dissolved in the patient’s body water (easily accomplished, since its H_2_O solubility is 52 g/L), this would contribute ~4 mOsm/kg to the total serum osmolality. Therefore, the ingested butoxypropanol would likely not account for the entire osmolal gap observed. Nevertheless, the absence of an elevated anion gap, hypocalcemia, and crystalluria argued against the osmolal gap occurring due to ethylene glycol exposure.

Other potential causes of this patient’s acute kidney injury that were considered but ruled out as unlikely include rhabdomyolysis (serum creatine phosphokinase too low), and acute tubular necrosis from shock (patient not hypotensive on arrival). Dehydration from repeated vomiting and diarrhea may have contributed to the acute kidney injury on admission; however, the initial BUN-to-creatinine ratio (BUN 22 mg/dl; creatinine 2.9 mg/dL) suggests intrinsic renal injury as a more likely cause.

## CONCLUSION

Despite widespread use, reports of acute toxicity from human exposures to the fungicide chlorothalonil are rare. In addition to the previously reported effects of gastrointestinal distress, oral ulceration, and a seizure, acute ingestion of chlorothalonil is very likely associated with reversible kidney injury. The patient reported here presented with acute kidney injury consistent with acute tubular necrosis, as demonstrated by elevated serum BUN and creatinine and granular casts in the urinary sediment, that resolved with IV hydration and supportive care.

## Figures and Tables

**Figure:**
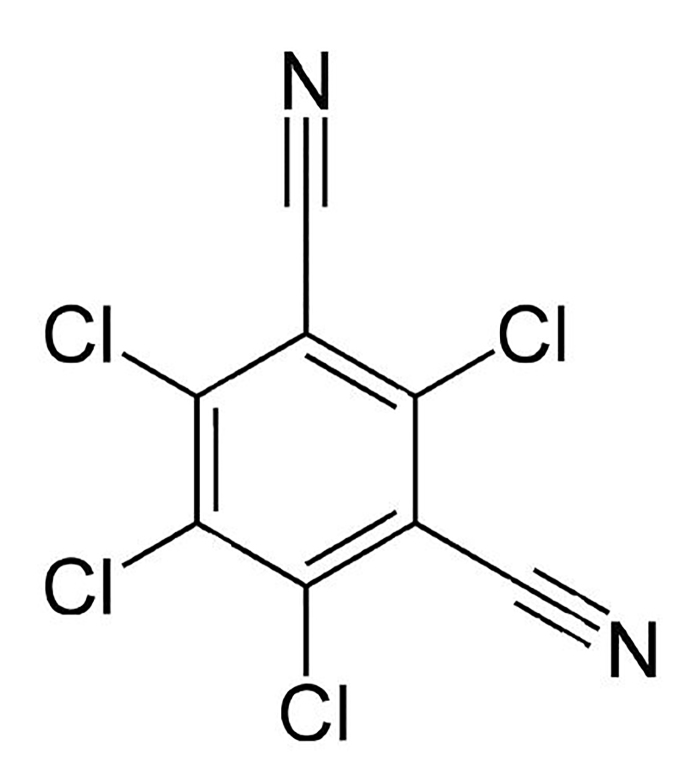
Chemical structure of chlorothalonil (2,4,5,6-tetrachloroisophthalonitrile), a broad-spectrum organochlorine fungicide widely used in agriculture
